# Supplementary material for: Comparison of Deep Learning Algorithms in Predicting Expert Assessments of Pain Scores during Surgical Operations Using Analgesia Nociception Index
Source: Sensors (Basel). 2022 Jul 23;22(15):5496. doi: 10.3390/s22155496 (PMC9330343; doi:10.3390/s22155496)
Supplement: Supplementary file 1 [file sensors-22-05496-s001.zip › sensors-1782526-supplementary.pdf]

Supplementary Materials

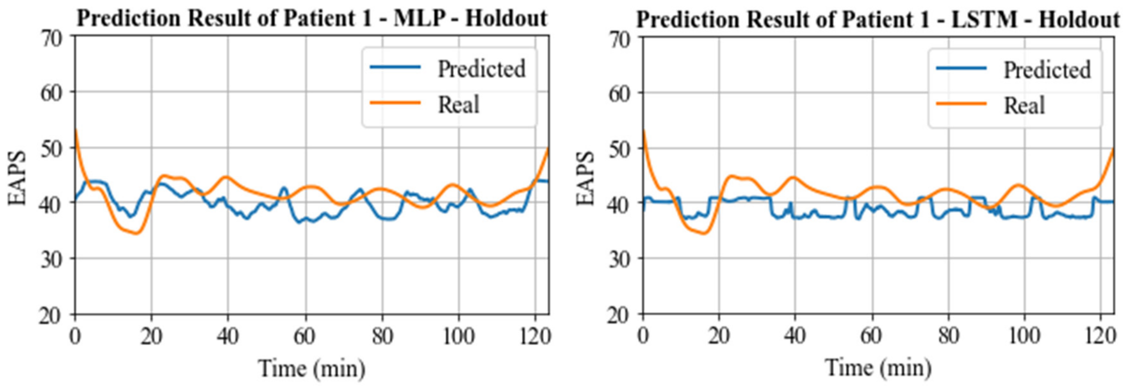

(a) Patient 1

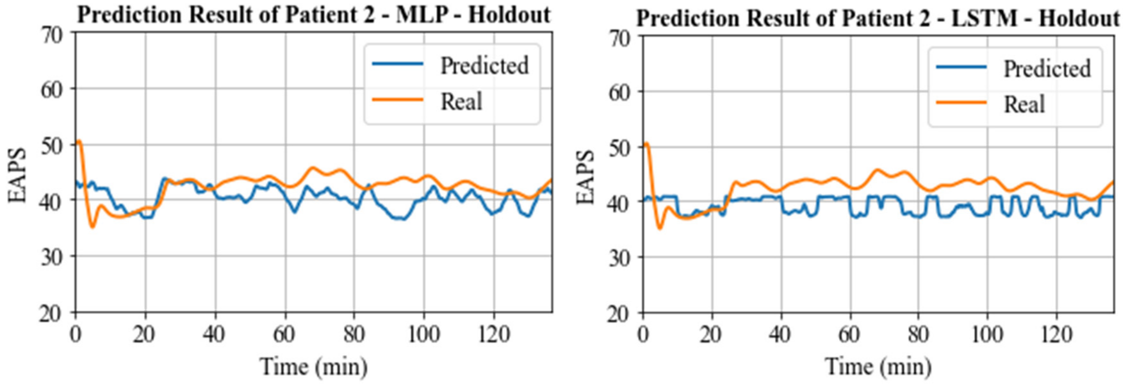

(b) Patient 2

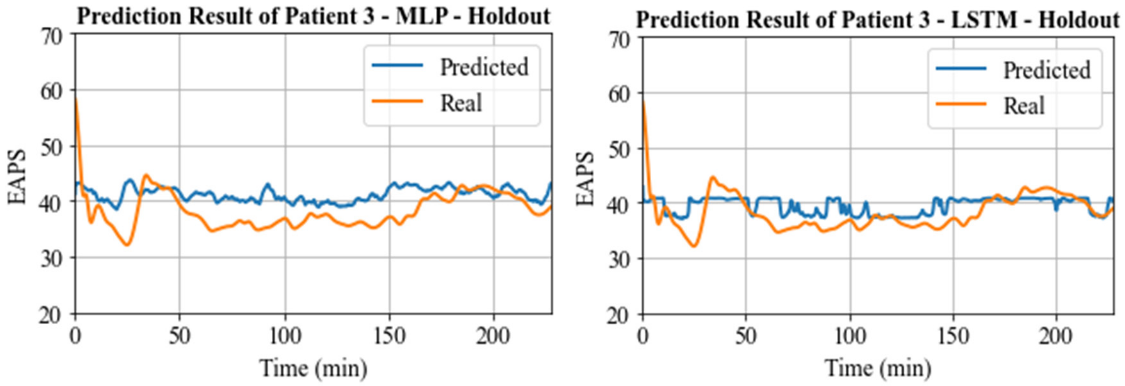

(c) Patient 3

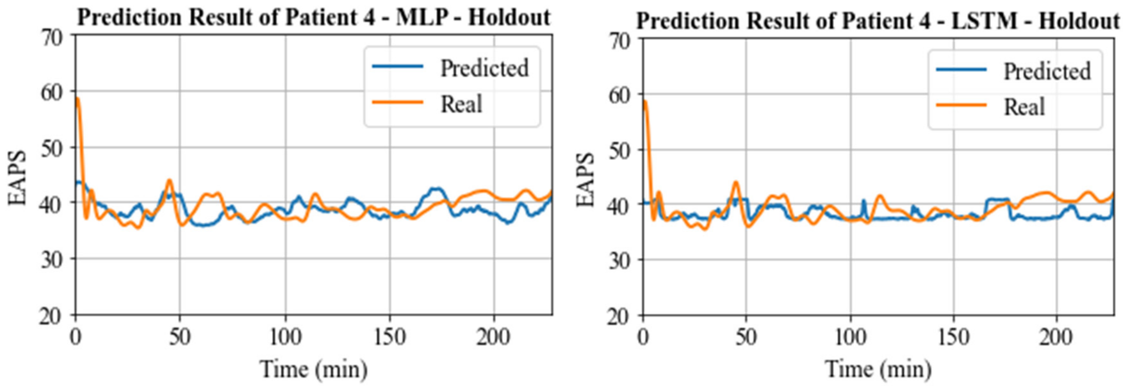

(d) Patient 4

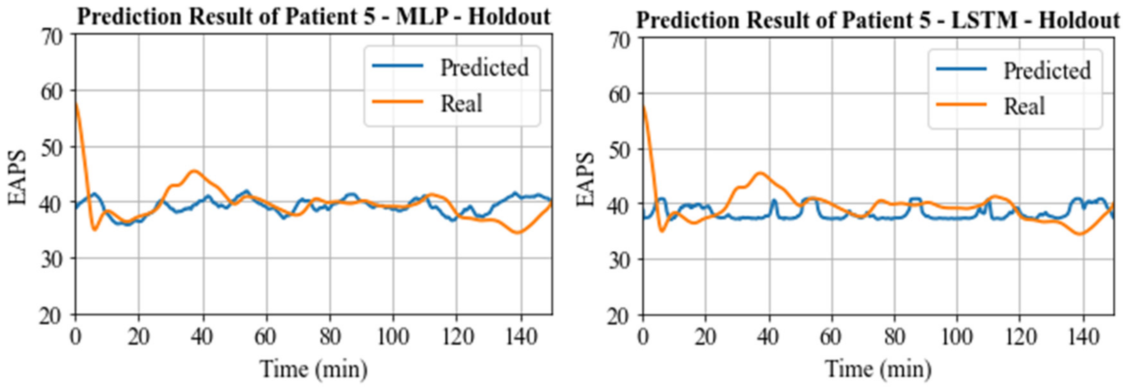

(e) Patient 5

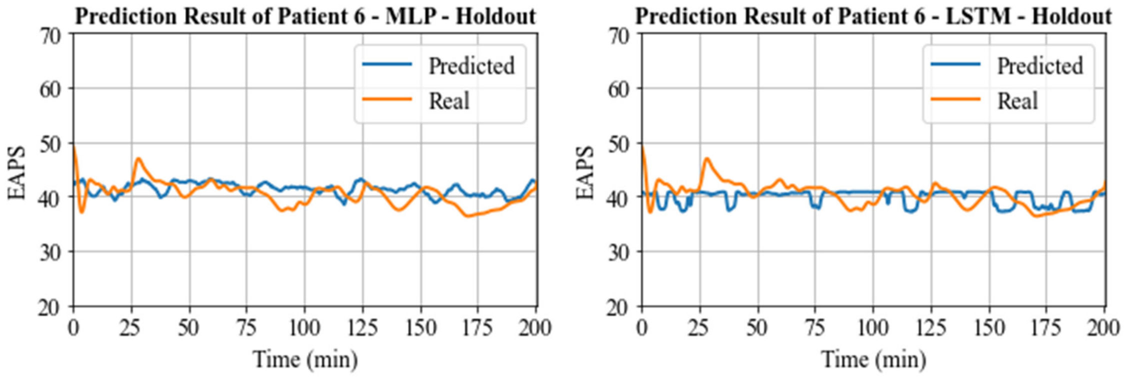

(f) Patient 6

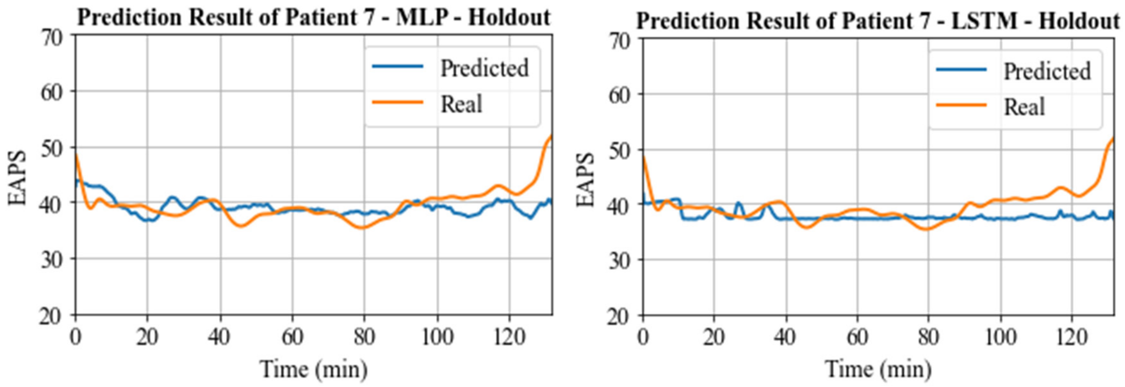

(g) Patient 7

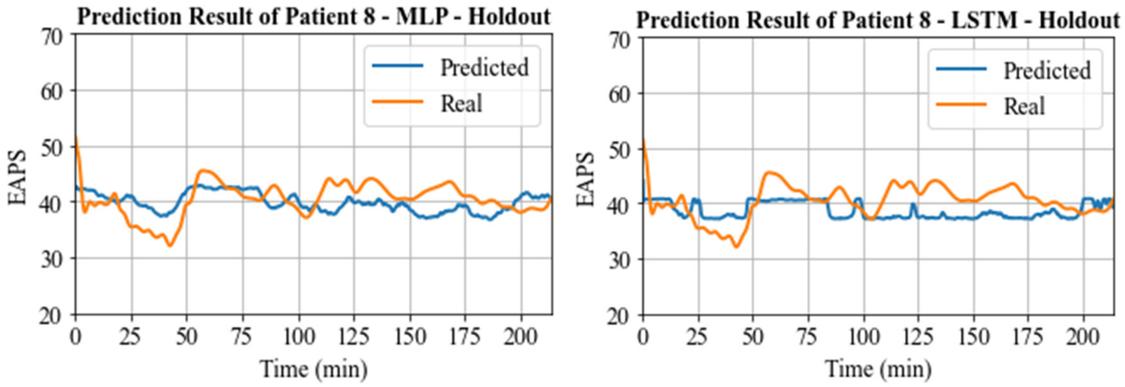

(h) Patient 8

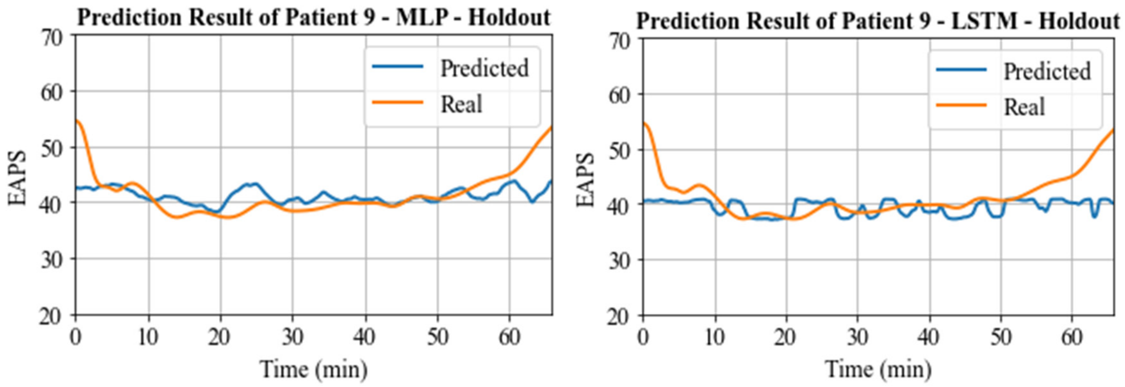

(i) Patient 9

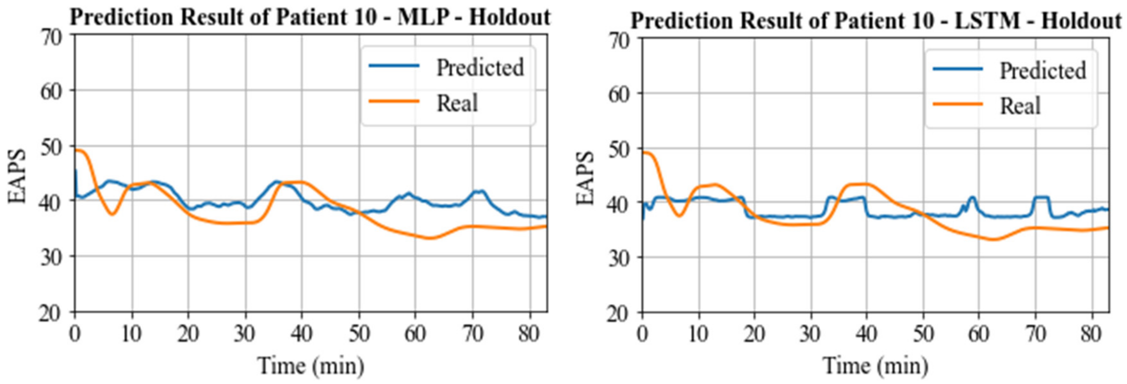

(j) Patient 10

Figure S1. Plots of Prediction Results from the MLP (Left Side) and the LSTM (Right Side) Models in the Holdout Method

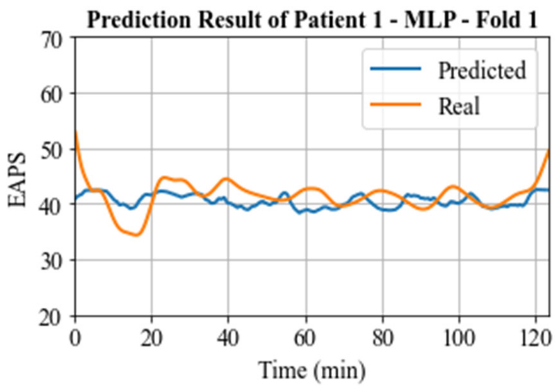

(a) Patient 1

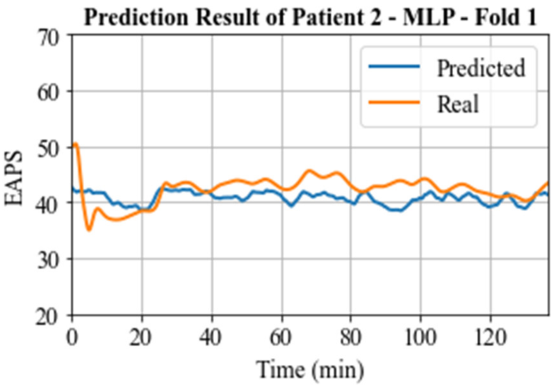

(b) Patient 2

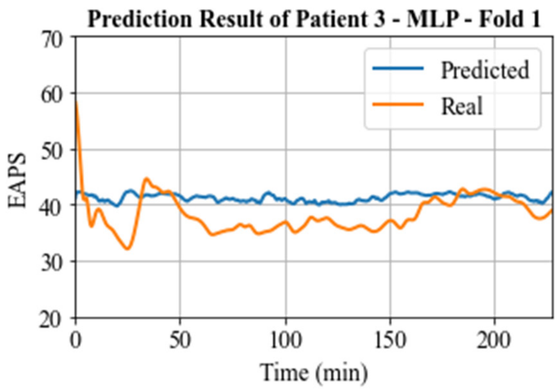

(c) Patient 3

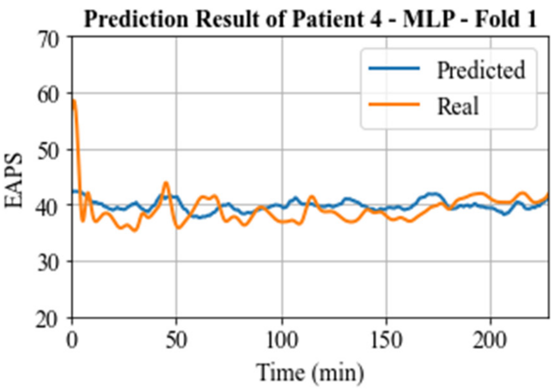

(d) Patient 4

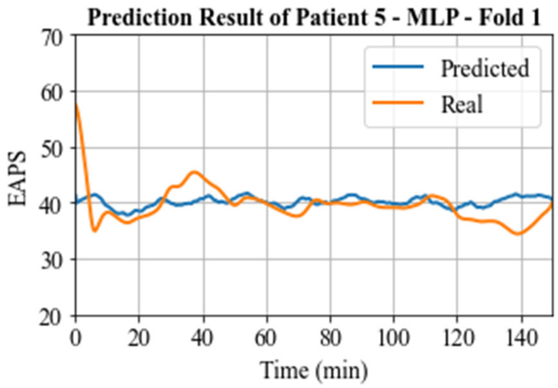

(e) Patient 5

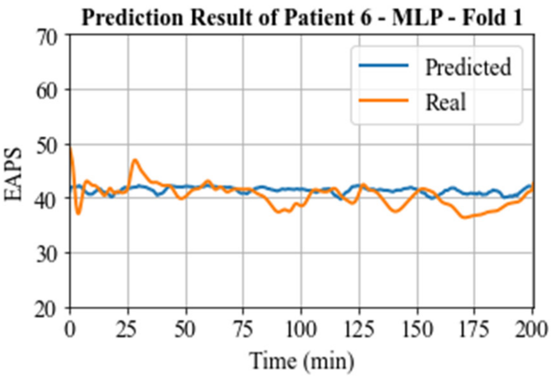

(f) Patient 6

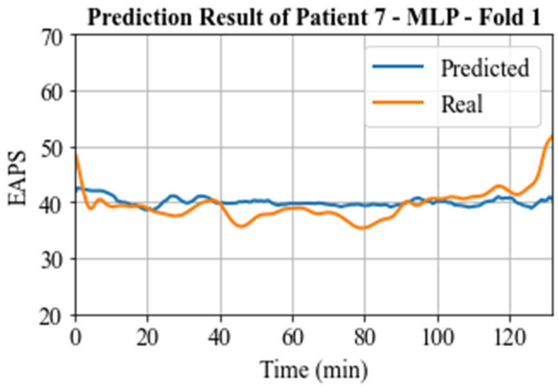

(g) Patient 7

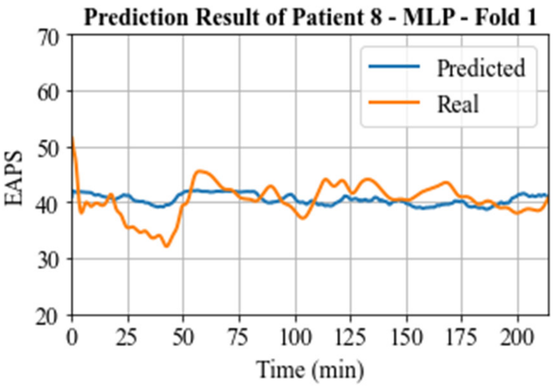

(h) Patient 8

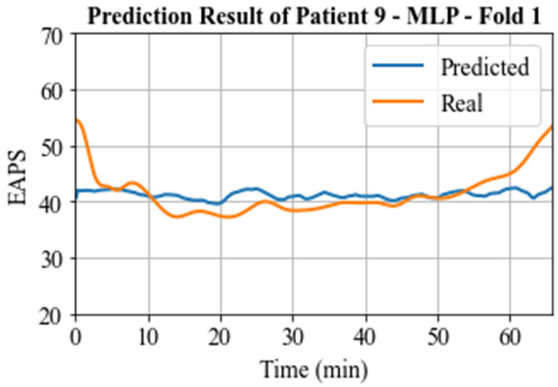

(i) Patient 9

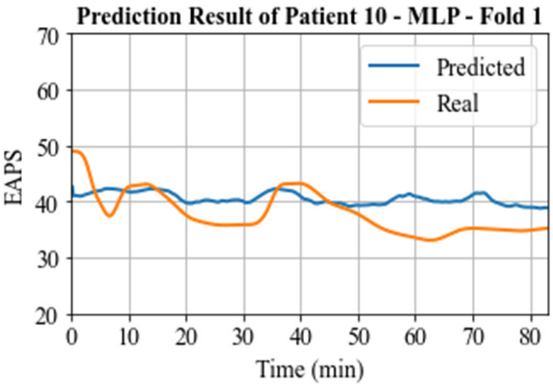

(j) Patient 10

Figure S2. Plots of Prediction Results from the First Fold MLP Model
